# Predicted Bacterial Interactions Affect *in Vivo* Microbial Colonization Dynamics in *Nematostella*

**DOI:** 10.3389/fmicb.2018.00728

**Published:** 2018-04-24

**Authors:** Hanna Domin, Yazmín H. Zurita-Gutiérrez, Marco Scotti, Jann Buttlar, Ute Hentschel Humeida, Sebastian Fraune

**Affiliations:** ^1^Zoological Institute, Christian-Albrechts-Universität zu Kiel, Kiel, Germany; ^2^RD3 Marine Microbiology, GEOMAR Helmholtz Centre for Ocean Research Kiel, Kiel, Germany; ^3^RD3 Experimental Ecology, GEOMAR Helmholtz Centre for Ocean Research Kiel, Kiel, Germany; ^4^Christian-Albrechts-Universität zu Kiel, Kiel, Germany

**Keywords:** correlation networks, bacteria–bacteria interactions, holobiont, host–microbe interactions, Cnidaria, metaorganism, resilience, community ecology

## Abstract

The maintenance and resilience of host-associated microbiota during development is a fundamental process influencing the fitness of many organisms. Several host properties were identified as influencing factors on bacterial colonization, including the innate immune system, mucus composition, and diet. In contrast, the importance of bacteria–bacteria interactions on host colonization is less understood. Here, we use bacterial abundance data of the marine model organism *Nematostella vectensis* to reconstruct potential bacteria–bacteria interactions through co-occurrence networks. The analysis indicates that bacteria–bacteria interactions are dynamic during host colonization and change according to the host’s developmental stage. To assess the predictive power of inferred interactions, we tested bacterial isolates with predicted cooperative or competitive behavior for their ability to influence bacterial recolonization dynamics. Within 3 days of recolonization, all tested bacterial isolates affected bacterial community structure, while only competitive bacteria increased bacterial diversity. Only 1 week after recolonization, almost no differences in bacterial community structure could be observed between control and treatments. These results show that predicted competitive bacteria can influence community structure for a short period of time, verifying the *in silico* predictions. However, within 1 week, the effects of the bacterial isolates are neutralized, indicating a high degree of resilience of the bacterial community.

## Introduction

Central for the ability to predict the rules determining the assemblage of host-associated microbial communities is the knowledge about the factors influencing their dynamics and stability. It is well established that extrinsic factors, like temperature (Fan et al., 2013; Mortzfeld et al., 2016), pH (Ribes et al., 2016), or pathogens (Agler et al., 2016), can influence the community membership. In addition, a number of studies describe host factors shaping the host-associated microbiota, e.g., the innate immune system (Vaishnava et al., 2008; Franzenburg et al., 2012, 2013), diet (David et al., 2014), or host mucus composition (Staubach et al., 2012; Kashyap et al., 2013). In contrast, less is known about how bacteria–bacteria interactions themselves affect the assemblage of host-associated bacterial communities. However, it is known that interactions within microbial communities can be complex ranging from cooperation to competition (Deines and Bosch, 2016). They can be influenced by diverse factors, like bacterial metabolism (Grosskopf and Soyer, 2014; Zelezniak et al., 2015), environmental factors, or spatial organization (Kim et al., 2008; Mitri and Foster, 2013).

Until recently, it was assumed that cooperative interactions within host-associated bacterial communities are the driving force for stability and productivity (reviewed in Deines and Bosch, 2016). However, this view was challenged by theoretical work, which is based on ecological network analysis. While cooperative communities are predicted to be highly productive for the short term and unstable for the long term, competitive communities tend to be more diverse and stable over time (Coyte et al., 2015). Although much progress has been made in characterizing host-associated bacterial communities, few data are available on ecological interactions within these communities *in vivo* and their impact on community stability and dynamics.

The marine sea anemone *Nematostella vectensis* is characterized by a stable associated bacterial community, which is dynamic in response to host development (Mortzfeld et al., 2016). Host development is defined by several life stages. Upon fertilization, the embryos develop into free swimming planula larvae within 1–3 days. After roughly 1 week, the larvae metamorphose into sessile primary polyps. Sexual maturity is reached after 3–6 months (Hand and Uhlinger, 1994). In a previous study, the establishment of the bacterial community was monitored from the early developmental stages up to the reproductive adults over the time course of more than 1 year (Mortzfeld et al., 2016). Using this comprehensive dataset, we inferred theoretical bacteria–bacteria interactions (Faust and Raes, 2012; Weiss et al., 2016) to determine bacteria with a distinct predicted motif. Using bacterial isolates possessing predicted competitive or cooperative interactions, we tested their impacts on the assemblage of the microbiota in recolonization experiments in juvenile animals. Our results show that predicted competitive bacteria can influence community diversity for a short period of time, verifying the *in silico* predictions. With this study, we confirm the significance of the predictions of co-occurrence networks by experimentally testing the predicted ecological role of bacterial interactions.

## Materials and Methods

### Inference of Bacteria–Bacteria Co-occurrence Networks

Network links were inferred using correlation analysis among 508 OTUs representing the relative bacterial abundance in *N. vectensis* (Mortzfeld et al., 2016). SparCC methodology (Friedman and Alm, 2012) was chosen as the inference method because it was explicitly designed for compositional (i.e., based on relative information) and sparse (with a small amount of non-zero values compared to the maximum possible) data, two key features displayed by the sequencing data used in our study. As the amount of significant correlations (pseudo *p*-value ≤ 0.05) was large, only the strongest correlations were considered for network construction and analysis (i.e., strong correlations are those exceeding 0.5 in absolute value).

### Correlation Significance

SparCC methodology assigns a pseudo *p*-value to each correlation through a bootstrap approach. The pseudo *p*-value represents the proportion of times a correlation from permutated datasets is at least as extreme as the observed “real” one (Friedman and Alm, 2012). To calculate the pseudo *p*-values, 1,000 permutated datasets with a two-sided distribution were used.

### Network Descriptors

The links in the co-occurrence networks can be either negative or positive. The value assigned to the interactions (i.e., interaction strength) ranges between -1 and +1, and the sign can provide proxies on the type of interaction (e.g., positive correlations can stand for cooperative activities, while negative correlations can indicate competition; see Shade et al., 2012). The number of nodes (the size of the network, which corresponds to the total number of OTUs; *N*), the number of links (the total number of significant correlations exceeding 0.5 in absolute value; *L*), the number of connected nodes (the OTUs with at least one interaction; *N*_C_), the density [the ratio between *L* and the maximum number of links that an undirected network can have: *L*_max_ = *N* (*N* - 1)/2; *D* = *L*/*L*_max_], the numbers and proportions of positive (*L*_P_, %*L*_P_) and negative (*L*_N_, %*L_N_*) links, the mean correlation values based on total (*m*_t_), positive (*m*_p_), or negative (*m*_n_) interactions, and the number of subnetworks (networks composed by isolated subsets of *N*, where the nodes of each subnetwork show no connections outside the subset; *n*_sub_) were taken as network descriptors. The degree (*d*) of the nodes was used as an indicator of centrality to identify the most important OTUs in the network (Wasserman and Faust, 1994). Thus, an OTU *i* was considered to be important when it had a high degree (*d*_i_ is large if the node *i* is directly linked to several OTUs) and most connections of the same sign (i.e., to discriminate among cooperators or competitors). Also the mean 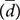 and the maximum (*d*_max_) degrees of the networks were calculated as global descriptors starting from single node values.

### Animal Culture

All experiments were carried out with juvenile polyps of *N. vectensis*. The adult animals of the laboratory culture were F1 offspring of CH2XCH6 individuals collected from the Rhode River in Maryland, United States (Hand and Uhlinger, 1992; Fritzenwanker and Technau, 2002). They were kept under constant, artificial conditions without substrate or light in *Nematostella* Medium (NM), which was adjusted to 18°C and 16‰ salinity with Red Sea Salt^®^ and Millipore H_2_O. Polyps were fed 2–3 times a week with first instar nauplius larvae of *Artemia salina* as prey (Ocean Nutrition Micro *Artemia* Cysts 430–3500 g, Coralsands, Wiesbaden, Germany).

### Antibiotic Treatment

The antibiotic treatment procedure was adapted after the protocol for germ-free (GF) *Hydra* polyps (Fraune et al., 2014). The juveniles were incubated without food supply for 4 weeks in sterile NM with an antibiotic cocktail of ampicillin, neomycin, streptomycin, spectinomycin, and rifampicin in a final concentration of 50 μg/mL each. The medium was changed every 2–3 days. After the 4 weeks, the polyps were transferred into antibiotic-free medium. The absence of cultivatable bacteria was checked at the end of the antibiotic treatment by plating homogenized polyps on Marine Bouillon (MB) plates. While a weak band was detected using specific 16S rRNA gene primers (27F and 338R), no recovery of bacterial colonization was observed based on PCR signal intensity and plating on MB during the course of the experiment in non-colonized animals.

### Recolonization

The bacterial load of larvae and juveniles was estimated by colony forming units (CFUs) of larvae and juveniles. Larvae (6 days old) and juveniles (8–10 tentacle stages) were homogenized and spread on MB plates. The plates were incubated at 18°C for 3 days before counting colonies. One smashed larva resulted in ∼200 colonies grown on MB plates and one smashed juvenile spread on an MB plate yielded ∼2,000 colonies. To ensure successful recolonization, the polyps were exposed to double the amount of their native microbiota (e.g., ∼4,000 colonies per juvenile). The bacterial isolates were grown to an OD600 of 0.2, spread out on MB plates, and counted in order to calculate the cell number.

Prior to recolonization, the juvenile polyps were treated with antibiotics for 4 weeks and remained in sterile antibiotic-free medium for 4 days before recolonization. The animals were starved during the whole experiment. For each recolonization treatment and replicate, 10 juvenile polyps were put into a 2 mL Eppendorf tube and filled up with 2 mL of one of the following solutions: (1) native larval bacteria; (2) native juvenile bacteria; or (3) a mix of native larval bacteria and one single bacterial isolate in overrepresentation. Complex bacterial mixtures were obtained by smashing whole larvae or juvenile polyps in sterile NM. The homogenates were centrifuged and the pellet washed twice in sterile NM. Samples were collected for the three types of treatments at two time points. Five replicates per treatment and time point were used and each replicate consisted of five pooled animals.

The juveniles were recolonized with a mix of native larval bacteria together with single isolates with the aim of adding the single isolates in a 1:3 ratio of larval bacteria to single isolates. By sequencing the 16S rRNA genes of inocula, we estimated the overrepresentation of all isolates. Although it was not possible to obtain any mix of larval bacteria and bacterial isolates with the 1:3 target ratio, the five selected OTUs were still overrepresented at the start of each treatment, i.e., at least 10-fold their initial abundance in the control. The fold change of each isolate was estimated by comparing the sequencing reads of control (bL) to treatment.

### Cultivation and Identification of Bacterial Isolates

Bacteria were isolated from *Nematostella* polyps by smashing single polyps in sterile NM and plating them on MB, LB, and R2A agar plates. After incubation at 18°C for 5 days, single CFUs were isolated and cultivated in liquid MB, LB, or R2A medium. The bacteria were identified by Sanger sequencing of the 16S rRNA gene. Stocks were stored in Roti-Store cryo vials (Carl Roth, Karlsruhe, Germany) or in 50% glycerol at -80°C. Bacterial isolates were grown and isolated in the following media: OTU194 (*Ruegeria* sp.) and OTU1209 (*Vibrio* sp.) grew in MB medium, OTU1325 (*Aeromonas* sp.) and OTU941 (*Pseudomonas* sp.) in LB medium, and OTU670 (*Acinetobacter* sp.) in R2A medium.

### DNA Extraction and 16S rRNA Sequencing

Before extraction, the animals were washed three times with 500 μL sterile filtered NM and frozen without liquid at -80°C until extraction. The gDNA was extracted from whole five animals per sample with the DNeasy^®^ Blood & Tissue Kit (Qiagen, Hilden, Germany) as described in the manufacturer’s protocol. DNA was eluted in 200 μL elution buffer. The eluate was frozen at -20°C until sequencing. For each sample, the hypervariable regions V1 and V2 of bacterial 16S rRNA genes were amplified. The forward primer (5′-AATGATACGGCGACCACCGAGATCTACAC XXXXXXXX TATGGTAATTGT AGAGTTTGATCCTGGCTCAG-3′) and reverse primer (5′-CAAGCAGAAGACGGCATACGAGAT XXXXXXXX AGTCAGTCAGCC TGCTGCCTCCCGTAGGAGT-3′) contained the Illumina Adaptor p5 (forward) and p7 (reverse). Both primers contain a unique 8 base index (index; designated as XXXXXXXX) to tag each PCR product. For the PCR, 100 ng of template DNA (measured with Qubit) were added to 25 μL PCR reactions, which were performed using Phusion^®^ Hot Start II DNA Polymerase (Finnzymes, Espoo, Finland). All dilutions were carried out using certified DNA-free PCR water (JT Baker). PCRs were conducted with the following cycling conditions (98°C – 30 s, 30 × [98°C – 9 s, 55°C – 60 s, 72°C – 90 s], 72°C – 10 min) and checked on a 1.5% agarose gel. The concentration of the amplicons was estimated using a Gel Doc^TM^ XR+ System coupled with Image Lab^TM^ Software (BioRad, Hercules, CA, United States) with 3 μL of O’GeneRulerTM 100 bp Plus DNA Ladder (Thermo Fisher Scientific, Inc., Waltham, MA, United States) as the internal standard for band intensity measurement. The samples of individual gels were pooled into approximately equimolar subpools as indicated by band intensity and measured with the Qubit dsDNA br Assay Kit (Life Technologies GmbH, Darmstadt, Germany). Subpools were mixed in an equimolar fashion and stored at -20°C until sequencing. Sequencing was performed on the Illumina MiSeq platform with v3 chemistry (Rausch et al., 2016). The raw data are deposited at the Sequence Read Archive (SRA) and available under the project ID PRJNA433067.

### Analyses of Bacterial Communities

The sequence analysis was conducted using the QIIME 1.9.0 package (Caporaso et al., 2010). Paired end reads were assembled using SeqPrep. Chimeric sequences were identified with Chimera Slayer (Haas et al., 2011). OTU picking was performed using the pick_open_reference_otus.py protocol with at least 97% identity per OTU and annotation was conducted with the UCLUST algorithm (**RRID**:SCR_011921; Edgar, 2010) against the GreenGenes database v13.8 (**RRID**:SCR_002830; DeSantis et al., 2006) implemented in QIIME. OTUs with less than 50 reads were removed from the dataset to avoid false positive OTUs that may originate from sequencing errors (Faith et al., 2013). The number of reads was normalized to 10,000 reads for the analysis. Alpha-diversity was calculated with the Chao1 metric implemented in QIIME using ten replicates of rarefication per sample. Beta-diversity was depicted in a PCoA by 100 jackknifed replicates using Bray–Curtis and weighted UniFrac metrics. For statistical analysis of clustering the method ADONIS was used.

## Results

### Bacteria–Bacteria Co-occurrence Networks During Host Development

**FIGURE 1:**
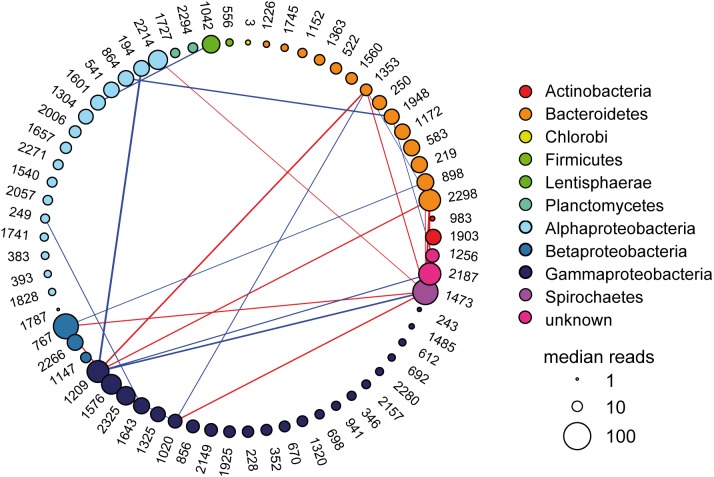
Microbial co-occurrence network among OTUs during the whole development of *N. vectensis*. Nodes (*N* = 66) are the OTUs involved in at least one strong interaction during the whole development or the three developmental stages; their color reflects taxonomic affiliation. The size of the nodes is proportional to the log_10_ of the median reads (relative abundance of the OTUs) along the whole development. OTUs are arranged by taxonomy and relative abundance. Links represent the interactions (i.e., significant co-occurrences; pseudo *p*-value ≤ 0.05) with absolute correlation values above 0.5. Red links are negative interactions, while blue links stand for positive interactions; the thickness of the links is proportional to the strength of the interactions.

**FIGURE 2:**
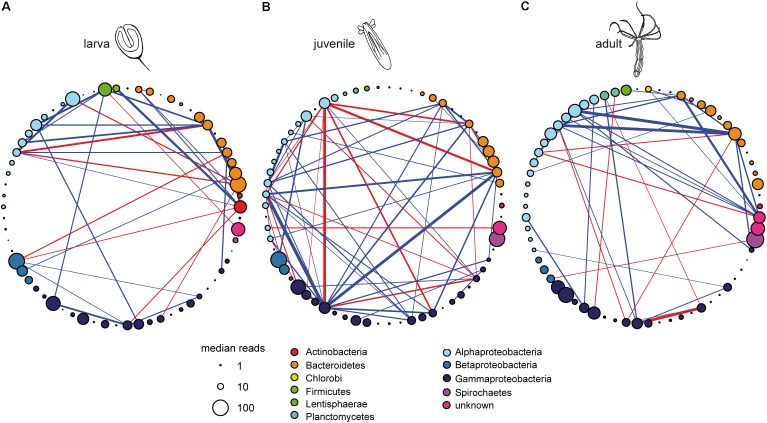
Microbial co-occurrence networks for **(A)** larval stage, **(B)** juvenile stage, and **(C)** adult polyps of *N. vectensis*. Nodes (*N* = 66) are the same as in **Figure 1** and Supplementary Figure S1 their order in the circular arrangement was preserved. OTU numbers are provided in Supplementary Figure S1. The colors inform about the taxonomic affiliation of the nodes. The size of the nodes is proportional to the relative abundance of the OTUs (measured as log_10_ of median reads) in each developmental stage. Links are significant correlations (pseudo *p*-value ≤ 0.05) with absolute values above 0.5; their color allows distinguishing among negative (red) and positive (blue) interactions, while the thickness is proportional to the strength.

To infer potential bacteria–bacteria interactions in the bacterial community of *N. vectensis*, network links were inferred using SparCC methodology (Friedman and Alm, 2012) to the relative abundance of 508 OTUs over the whole ontogeny (Mortzfeld et al., 2016). Using bacterial abundance data, network correlations were inferred from: (1) all sampling time points together, leading to the representation of the most important interactions along the whole development of the animal and (2) the three developmental stages separately, which characterize the most relevant correlations during each developmental stage. For the construction of the co-occurrence networks, the strongest significant interactions (i.e., those with pseudo *p*-value ≤ 0.05 and an absolute correlation value larger than 0.5) in each of the datasets were selected. A list of 66 nodes (*N* = 66), representing 66 bacterial OTUs, was obtained from the union of all OTUs that were found at least once in one of the four datasets of the significant and strong correlations. Using these 66 nodes, the four co-occurrence networks were constructed. **Figure 1** is the co-occurrence network along the whole developmental process of *N. vectensis.* **Figure 2** shows the co-occurrence networks for each developmental stage. The OTUs were arranged by taxonomy and relative abundance computed for the whole development (**Figure 1**) and the same order is preserved in **Figure 2** OTU numbers are provided in **Figure 1** and Supplementary Figure S1 but showing the relative abundance of OTUs at each developmental stage.

None of the constructed networks has more than 56 interactions (*L* = 56) or involves more than 29 OTUs (*N*_C_ ≤ 29), resulting in a low density across all networks (**Table 1**). All networks have more positive than negative interactions (*L*_P_ > *L*_N_), which is reflected in the mean correlation values calculated considering the total set of links (**Table 1**). All networks are composed of two or more subnetworks, but this could be a consequence of the chosen correlation cut-off rather than a biological property. The four networks together have 145 interactions and only one shared interaction between different developmental stages (i.e., the interaction OTU1601–OTU1657 is present in both larval and adult stages).

**Table 1:** Network descriptors used to characterize the properties of the correlation networks. Indices were calculated for both the whole development network (i.e., based on all correlations among OTUs, irrespective of the various stages of polyp growth) and the networks that refer to three developmental stages (i.e., larva, juvenile, and adult). All networks are composed of the same 66 OTUs (*N* = 66).

Descriptors	Whole development	Larva	Juvenile	Adult
Number of links (*L*)	22	35	56	37
Number of connected nodes (*N*_C_)	20	25	29	29
Density of the network (*D*)	0.010	0.016	0.026	0.017
Number of positive links (*L*_P_)	12	25	39	27
Number of negative links (*L*_N_)	10	10	17	10
Proportion of positive links (%*L*_P_)	0.545	0.714	0.696	0.730
Proportion of negative links (%*L*_N_)	0.455	0.286	0.304	0.270
Mean of total correlations (*m*_t_)	0.045	0.250	0.218	0.260
Mean of positive correlations (*m*_p_)	0.535	0.569	0.559	0.559
Mean of negative correlations (*m*_n_)	-0.544	-0.548	-0.565	-0.547
Number of subnetworks (*n*_sub_)	4	2	6	5
Mean degree 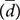	2.200	2.800	3.862	2.552
Maximum degree (*d*_max_)	7	8	14	7
OTUs with maximum degree	1473	1903	1643	1948, 1601, 1256

The co-occurrence network spanning the whole host development (**Figure 1**) has the lowest number of connected nodes (*N_C_* = 20; **Table 1**). Here, a spirochaete bacterium (OTU1473) has the highest degree of links indicating a potential role as organizer along the whole development of *N. vectensis* (Supplementary Table S1 and **Figure 1**). Interestingly, when analyzing the different developmental phases separately, the structure of the interactions (**Figure 2**) and the degree of the nodes (Supplementary Table S1) vary during animal development. Thus, the set of nodes with the highest degrees (i.e., OTUs with the higher number of direct links in the co-occurrence network; **Table 1**) is also modified, which reflects how the importance of the various phylogenetic groups changes through development. At the larval stage, the strongest correlations are mainly found between Actinobacteria, Bacteroidetes, Lentisphaerae, and Alphaproteobacteria (**Figure 2A**), but these links change during the onset of development. During the juvenile stage, Gammaproteobacteria become greatly important, interacting mainly with Alphaproteobacteria and Bacteroidetes (**Figure 2B**). However, at the adult stage, almost all interactions are between Alphaproteobacteria, Bacteroidetes, and an unknown taxon (**Figure 2C**). While at the larval stage, the bacterium with the highest degree belongs to Actinobacteria (OTU1903), at the juvenile stage, it is replaced by a Gammaproteobacterium (OTU1643). At the adult stage, three different bacteria are the most connected: one bacterium from the Bacteroidetes (OTU1948), one from the Alphaproteobacteria (OTU1601), and one unknown bacterium (OTU1256; Supplementary Table S1). Interestingly, the network constructed from the bacterial data of juvenile animals shows the highest number of links (*L* = 56; **Table 1**). This suggests that in this developmental phase of the animal, the bacteria–bacteria interactions may be of greater importance for shaping the bacterial community composition than during the two other developmental phases.

### Experimental Testing of Predicted Bacteria–Bacteria Interactions

**FIGURE 3:**
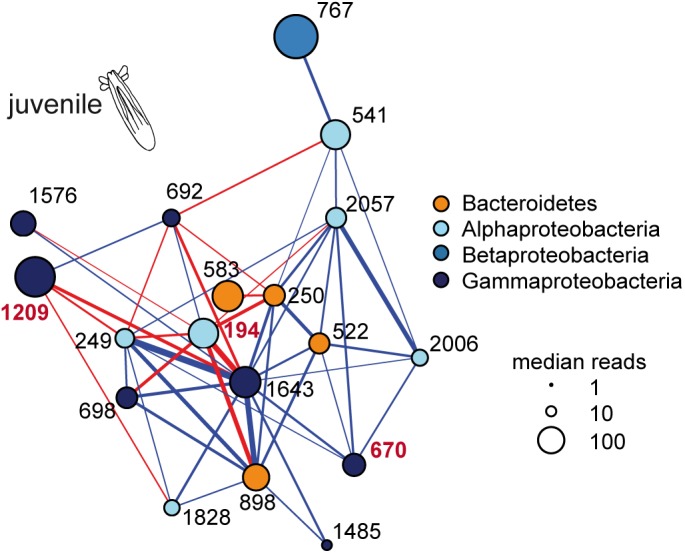
Dominant microbial co-occurrence subnetwork in juvenile polyps. The colors inform about the taxonomic affiliation of the nodes. The size of the nodes is proportional to the relative abundance of the OTUs (measured as log_10_ of median reads) in juvenile polyps. Links are significant correlations (pseudo *p*-value ≤ 0.05) with absolute values above 0.5; their color allows distinguishing among negative (red) and positive (blue) interactions, while the thickness is proportional to the strength. OTUs with representative isolates available are labeled in red.

In order to test the role of predicted bacteria–bacteria interactions in the assemblage of the juvenile microbiota *in vivo*, a cultivation approach of bacteria colonizing juvenile polyps was performed. Of all isolates, 13 bacterial strains were present within the 66 OTUs of the co-occurrence networks (**Table 2**). Nine bacterial strains belong to the Gammaproteobacteria, three bacterial strains belong to the Alphaproteobacteria, and one belongs to the Bacteroidetes. Within these 13 bacterial isolates, only three strains have more than one correlation within the co-occurrence network of juvenile animals (**Table 2**) and form part of the same subnetwork (**Figure 3**). The bacterial isolates representing OTU194 *(Ruegeria* sp.) and OTU1209 (*Vibrio* sp.) are characterized by mainly negative correlations and therefore may act as competitive bacteria. Both isolates belong to the group of most abundant colonizers in juvenile polyps (**Figures 2**, **3**), while in the bacterial community of larvae, they are underrepresented (Mortzfeld et al., 2016). In contrast, the isolate representing OTU670 (*Acinetobacter* sp.) exerts mainly positive correlations, thus seeming to be a cooperative bacterium (**Figure 3** and **Table 2**).

**Table 2:** Bacterial strains cultivated from juvenile polyps of *N. vectensis*.

#OTU	Bacterium	Consensus lineage	Clone	Correlations in juvenile network	
				Positive	Negative
1304	*Kiloniella* sp.	Alphaproteobacteria; Kiloniellales; Kiloniellaceae	JB_90	1	0
194	*Ruegeria* sp.	Alphaproteobacteria; Rhodobacterales; Rhodobacteraceae	JB_30	1	6
1657	*Loktanella* sp.	Alphaproteobacteria; Rhodobacterales; Rhodobacteraceae	JB_36	0	0
2298	*Flavobacterium* sp.	Bacteroidetes; Flavobacteriia; Flavobacteriales; Flavobacteriaceae	JB_91	1	0
1325	*Aeromonas* sp.	Gammaproteobacteria; Aeromonadales	JB_15	0	0
2280	*Marinobacter* sp.	Gammaproteobacteria; Alteromonadales; Alteromonadaceae	JB_35	0	0
1320	*Alteromonas* sp.	Gammaproteobacteria; Alteromonadales; Alteromonadaceae	JB_27	0	0
670	*Acinetobacter* sp.	Gammaproteobacteria; Pseudomonadales; Moraxellaceae	JB_10	5	0
941	*Pseudomonas* sp.	Gammaproteobacteria; Pseudomonadales; Pseudomonadaceae	JB_53	0	0
1576	*Francisella* sp.	Gammaproteobacteria; Thiotrichales; Francisellaceae	JB_85	1	0
1209	*Vibrio* sp.	Gammaproteobacteria; Vibrionales	JB_14	1	3
243	*Vibrio* sp.	Gammaproteobacteria; Vibrionales; Vibrionaceae	JB_01	0	0
2325	*Vibrio* sp.	Gammaproteobacteria; Vibrionales; Vibrionaceae	JB_81	0	0

**FIGURE 4:**
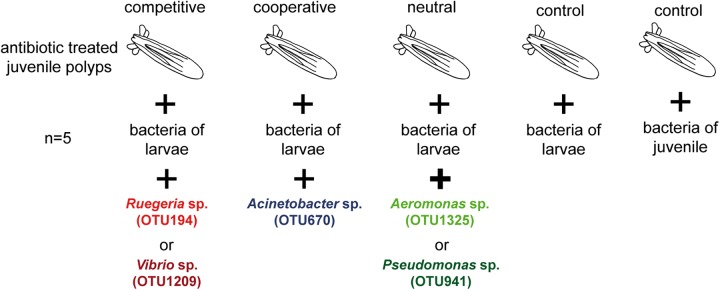
Experimental setup for the recolonization experiments. For each treatment, juvenile polyps were treated with antibiotics and then recolonized with different bacterial inocula. For recolonizations with competitive, cooperative, or neutral bacteria, the selected OTUs were mixed with bacteria of larvae. For the two controls, antibiotic treated juveniles were recolonized with bacteria of larvae or juveniles alone.

Using these three bacterial isolates, it was tested if predicted bacteria–bacteria interactions influence the assemblage of the juvenile microbiota *in vivo*. Therefore, the experiments with antibiotic-treated juvenile polyps were conducted by recolonizing with: (1) larval bacteria; (2) juvenile bacteria; and (3) larval bacteria mixed with single bacterial isolates in excess (**Figure 4**). Two isolates without any correlations at the juvenile stage, OTU1325 (*Aeromonas* sp.) and OTU941 (*Pseudomonas* sp.), were selected as controls (Supplementary Table S1). The recolonization with larval bacteria was chosen as the tested bacterial isolates are not overrepresented in this bacterial community and this allows their overrepresentation in the recolonization experiments. All treatments were conducted with five independent replicates, sampled at 3- and 7-day post-recolonization (dpr) and analyzed by 16S rRNA gene profiling.

### Recolonization of Juvenile Polyps With Larval and Juvenile Bacteria

**FIGURE 5:**
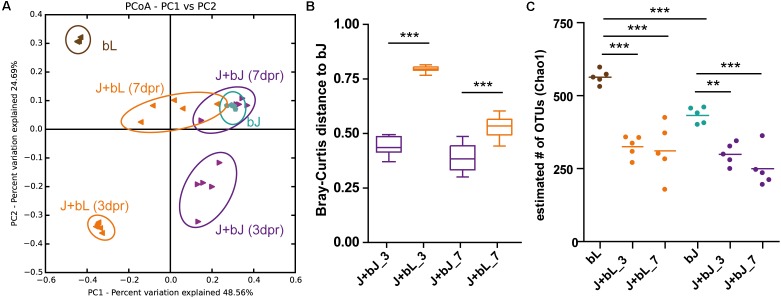
Juvenile polyps recolonize differentially with larvae (bL) or juvenile bacteria (bJ). **(A)** Bacterial communities were clustered using PCoA of the Bray–Curtis distance matrix. The percent variation explained by the principal coordinates is indicated on the axes. bL, source bacteria of larvae; bJ, source bacteria of juvenile polyps; J+bL, bacterial community of polyps recolonized with bL after 3dpr and 7dpr; J+bJ, bacterial community of polyps recolonized with bJ after 3dpr and 7dpr. **(B)** Bray–Curtis distances to bJ after 3dpr and 7dpr. **(C)** Estimated number (Chao1) of OTUs of the source communities and recolonization communities. Statistical analysis was conducted using analysis of variance (ANOVA; ^∗∗^*p* < 0.01, ^∗∗∗^*p* < 0.001).

Juvenile polyps which were inoculated with either juvenile (bJ) or larval bacteria (bL) showed a different community composition after 3dpr in comparison to the inocula and to each other (**Figure 5A**, ADONIS *R*^2^ = 0.95, *p* < 0.001). After 7 days of recolonization, both bacterial communities shifted back in the direction of the native bacterial situation characterizing juvenile polyps. The animals recolonized with bacteria of juveniles resembled hereby the native situation significantly better than animals recolonized with bacteria from larvae (**Figure 5B**). Similar results were obtained when calculating weighted UniFrac distances instead of Bray-Curtis distances (Supplementary Figure S2). In contrast, the recolonized animals showed no difference in their bacterial alpha-diversity, even though they were recolonized with bacterial inocula that differed significantly in their alpha-diversity (**Figure 5C**).

These results indicate that juvenile polyps can be recolonized with different source bacterial communities, but over time they develop back to the native juvenile community composition. However, only around 70% of the total bacterial diversity of juvenile polyps (bJ) could be restored within 7dpr (**Figure 5C**), independently of the alpha-diversity of the bacterial inoculum.

### Influence of Bacterial Isolates on Colonization Process

Before testing the effect of bacterial isolates on the composition assemblage in juvenile polyps, it was first checked if the overrepresented bacterial isolates are able to colonize the polyp. Over the course of the experiment, all five isolates remained overrepresented (Supplementary Figure S3). At 3dpr, the isolates were overrepresented between 3- and 27-fold (Supplementary Figure S3B). While both competitive bacteria (OTU194; *Ruegeria* sp. and OTU1209; *Vibrio* sp.) showed the highest initial colonization efficiency, one of the neutral isolates (OTU1325; *Aeromonas* sp.) recolonized with the lowest efficiency (Supplementary Figure S3B). At 7dpr, all bacterial isolates showed a similar overrepresentation of two to fivefold compared to the control (Supplementary Figure S3C). Therefore, it was possible to recolonize the juvenile polyps with an overrepresentation of bacterial isolates.

**FIGURE 6:**
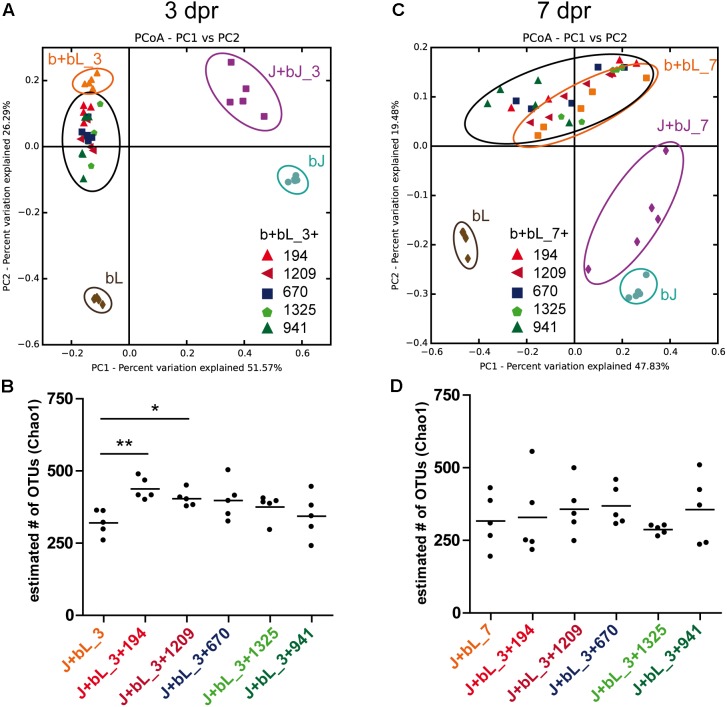
Recolonization patterns in the presence of selected isolates. Bacterial communities at 3dpr **(A)** and 7dpr **(C)** were clustered using PCoA of the Bray–Curtis distance matrix. The percent variation explained by the principal coordinates is indicated at the axes. bL, source bacteria of larvae; bJ, source bacteria of juvenile polyps; J+bL, bacterial community of polyps recolonized with bL after 3dpr and 7dpr; J+bJ, bacterial community of polyps recolonized with bJ after 3dpr and 7dpr; b+bL+isolates, bacterial community of polyps recolonized with bL and one of the selected isolates. Estimated number (Chao1) of OTUs after 3dpr **(B)** and 7dpr **(D)**. Statistical analysis was conducted using analysis of variance (ANOVA; ^∗^*p* < 0.05, ^∗∗^*p* < 0.01, *n* = 5).

To test for the effect of bacterial isolates on bacterial community assemblage in juvenile polyps, the colonization dynamics with isolates were compared to the control colonization without isolates. At 3dpr, the community composition was significantly affected by the addition of all five different isolates compared to the control (Supplementary Figure S4A). Surprisingly all isolates, cooperative (OTU670; *Acinetobacter* sp.), competitive (OTU194; *Ruegeria* sp. and OTU1209; *Vibrio* sp.), or neutral (OTU1325; *Aeromonas* sp. and OTU941; *Pseudomonas* sp.), shifted the community composition in a similar pattern (**Figure 6A**). Additionally, the distances between juvenile bacteria and recolonized juvenile polyps became significantly smaller if bacterial isolates were added (Supplementary Figure S4A), indicating a slightly better reconstitution of the original juvenile microbiota in the presence of the isolates. Moreover, the competitive bacteria (OTU194; *Ruegeria* sp. and OTU1209; *Vibrio* sp.) caused a significantly greater alpha-diversity compared to the control; in contrast, cooperative and neutral isolates had no effect on the alpha-diversity of the bacterial community (**Figure 6B**).

However, the effect of the isolates on the Bray–Curtis distances (**Figure 6C** and Supplementary Figure S4B) and the alpha-diversity (**Figure 6D**) vanished after 7dpr. Therefore, all bacteria caused only temporary shifts in the community composition and only competitive bacteria were able to induce a significant but temporary increase in alpha-diversity.

## Discussion

Co-occurrence networks were constructed to quantify the importance of specific bacteria based on community-level interactions (Faust and Raes, 2012). The goal was to focus on the co-occurrence networks to infer the ecological role of the bacteria (i.e., cooperation and competition) and identify the hubs (i.e., bacteria with many direct connections of the same sign). The reestablishment of the whole bacterial community in the presence of cooperative, competitive, or neutral bacteria was tested with *in vivo* recolonization experiments. The bacteria with strongest predicted interactions changed the community composition during the early recolonization steps of *N. vectensis*, but only the communities inoculated with competitive bacteria exhibited a significant but temporary increase in alpha-diversity. Our study shows that co-occurrence network inference can be used to retrieve ecologically relevant interactions.

The network approach allows identifying the most important bacteria by their potential role in the community rather than solely relying on their relative abundance (e.g., Jordán et al., 2015). In our work, the degree of the nodes (OTUs) was used to study the direct effects of the bacteria in the community (Scotti and Jordán, 2010), under the assumption that co-occurrence networks can be informative of ecological processes. While at large phylogenetic levels, the abundance can still be a good descriptor of the microbial community associated to *N. vectensis* (Mortzfeld et al., 2016), the most abundant OTUs are not always those displaying the higher number of links (see **Figures 1**, **2**). Network analysis suggested potentially important bacteria and enabled designing *in vivo* experiments to test whether the predicted interactions are ecologically relevant.

Generalized Lotka–Volterra equations were previously applied to predict interactions in microbial communities, and the validity of model results was confirmed by culture experiments (Mounier et al., 2008). However, studies based on dynamical modeling routinely involve only a small number of species, and the validation of network inference (e.g., based on 16S rRNA sequencing data) with culture experiments is in its infancy (Faust and Raes, 2012). The novelty of our study stems from the ability to culture single bacterial isolates, representing certain OTUs, which allows experimental testing of their ecological roles predicted by analysis of co-occurrence networks.

The microbial networks, inferred using the bacterial data from larvae, juvenile, and adult polyps, demonstrate that bacterial interactions during host development are highly dynamic. On the one hand, aspects determining changes in the bacterial networks might be linked to physiological and immunological factors of the host that are remodeled during development as shown during metamorphosis in amphibians (Rollins-Smith, 1998; Faszewski et al., 2008) and insects (Vigneron et al., 2014). Especially, effector molecules of the innate immune system like AMPs (Salzman et al., 2010; Login et al., 2011; Franzenburg et al., 2013; Mukherjee et al., 2014) or the provision of selective nutrients by the host (Ley et al., 2006) may directly influence the bacterial interactions. In addition, the specific composition of complex carbohydrates on the boundary between epithelium and environment may have a huge impact on individual bacterial fitness and interactions between bacterial species (Kashyap et al., 2013; Pickard et al., 2014). On the other hand, observed changes within the bacterial interactions could be explained by successions driven by ecological bacterial interactions alone. Studying the succession of plant colonization of new habitats was part of ecological research for a long time already, but recently this approach also gained popularity to study successional patterns of microbial communities (Fierer et al., 2010). It was shown that microbial community successions in a host are accompanied by changes in the metabolic potential, adapting to environmental changes like diet (Koenig et al., 2011), but are also predictable after infection and recovery (David et al., 2014). However, the changes in microbial succession and metabolic potential also occur in the absence of a host, leaving these successions exclusively to ecological interactions between bacteria alone (Datta et al., 2016).

In the experiment, we show that the early recolonization dynamics depend on the initial bacterial inoculum, but after 7dpr all recolonizations result in a similar bacterial community composition (**Figures 5**, **6**). Three days after recolonization, the community composition observed for all treatments (i.e., those inoculated with cooperative, competitive, or neutral OTUs) was significantly different from both the native larval (bL) and the native juvenile (bJ) bacteria (**Figure 6**). Nevertheless, 7dpr all treatments resembled more the native microbiota of juveniles than the larval source used to assemble the communities (**Figure 5**). This process was more efficient when juveniles were recolonized with juvenile microbiota rather than with bacteria extracted from larvae. Even when starting from different initial conditions, all recolonization treatments that included isolates followed recolonization paths that were similar to that of native larval bacteria. Recolonization with competitive, cooperative, or neutral bacteria always developed toward attaining the native juvenile bacterial state, thus showing the resilience of the system to perturbations. One explanation, why even neutral bacteria showed an effect on the assembly of the community, could be that the neutral bacteria were chosen based on network inference (e.g., the decision of considering strong correlations as those with absolute values above the 0.5 threshold, or the use of the SparCC algorithm for correlation detection). Although neutral bacteria do not present strong correlations in the larval and juvenile networks, they still have the potential to influence the community during initial establishment of the community or the later development of the host (**Figure 2**).

The convergence of all communities toward the native juvenile bacterial state shows that the initial composition is crucial for the stability of the system. The tested communities in our experiment showed resilience irrespective of the interaction strategy of the OTUs added in excess. Although the interaction mode of overrepresented OTUs does not alter the long-term equilibrium of the community, the competitors are the only OTUs challenging the stability of the system. As described in the literature (Czaran et al., 2002; Coyte et al., 2015), the addition of competitive OTUs significantly increased community diversity, even though such an effect was transient.

Competitive interactions between members of the bacterial community are expected to increase community diversity (Czaran et al., 2002; Coyte et al., 2015), spatial structure (Kim et al., 2008), stability (Kelsic et al., 2015), and functioning (Wei et al., 2015). After 7dpr, all communities have transited to a more stable composition, as the number of OTUs is almost the same among treatments (**Figure 6**) and overrepresented OTUs declined. In our recolonization experiment, mainly the spatial structure got abrogated by the antibiotic treatment and homogenization of the inocula. While with our experiment, we cannot assess the spatial structure or the functioning of the community, we can clearly see that only competitive bacteria increase community diversity, which is predicted by ecological theory (Coyte et al., 2015). The temporal increase in alpha-diversity could be explained by the fact that during the initial phase, the spatial structure of the bacterial community is not yet reestablished. In this initial phase bacteria can exert contact-dependent competition, which is particularly relevant in the treatments with overrepresented competitive bacteria, leading those communities to higher diversity. With the reestablishment of spatial structure, contact-dependent competition might be less pronounced. This is often described in literature as a real-life game of “rock-paper-scissors” (Kerr et al., 2002; Reichenbach et al., 2007), in which coexistence of competing communities is ensured by local interaction and dispersal (van Nouhuys and Hanski, 2005).

Neither the larval nor the juvenile bacterial communities are the final state of the system. Both are transient configurations from which the adult stable community develops (Fieth et al., 2016; Mortzfeld et al., 2016). Although stability has been described in marine ecosystems for microbial communities associated to various host taxa (Schmitt et al., 2012; Hester et al., 2016), there are examples (i.e., microbiota communities of corals) that do not present high resilience to perturbations (Rosenberg et al., 2009; Pogoreutz et al., 2018). Previous research has shown that environmental perturbations trigger slight changes in the composition of *N. vectensis* microbiota (Mortzfeld et al., 2016), but these effects were minor compared to the ones associated to the host development. Therefore, it is possible that the bacterial community associated to *N. vectensis* is able to buffer internal shifts such as the overrepresentation of single members of the community, as was simulated with our experiment.

Our study cannot exclude that host–bacteria interactions played a role in the succession of the microbial community, like the innate immune system (Franzenburg et al., 2012, 2013), spatial restriction (Kim et al., 2008; Mark Welch et al., 2017), or diet (David et al., 2014). Therefore, further investigations are needed to understand whether bacteria–bacteria interactions, host–bacteria interactions, or both modulate the resilience of the bacterial community. In the same way, we cannot discard that working with the strongest inferred correlations could mask some network properties (e.g., network connectivity and degree of each node) of particular relevance when choosing an OTU to implement an experiment. With the increasing number of isolates, exploration of other network properties or centrality measurements might be possible, and we could even gain the capacity to study only a few interactions at a time in a synthetic community approach (Bodenhausen et al., 2014).

## Conclusion

The aim of this study was to experimentally show that co-occurrence networks infer ecologically relevant interactions. The recolonization treatments that included competitive bacteria resulted in increased alpha-diversity compared to treatments with cooperative or neutral OTUs and controls. Although the shift in community composition and diversity was short term (i.e., the effects vanished after 7 days), our results match the expectation of ecological theory for competitive players being able to increase the alpha-diversity (Coyte et al., 2015). This study provides experimental evidence about the ecological relevance of inferred correlations in microbial communities and is the first step to establish the marine sea anemone *N. vectensis* as an experimental model to test theoretical predictions in host-associated microbial communities.

## Author Contributions

HD, YZ-G, MS, UHH, and SF contributed to conception and design of the study. HD, YZ-G, and JB performed the research. HD, YZ-G, and SF performed the statistical analysis. HD, YZ-G, MS, and SF wrote the first draft of the manuscript. All authors contributed to manuscript revision and read and approved the submitted version.

## Conflict of Interest Statement

The authors declare that the research was conducted in the absence of any commercial or financial relationships that could be construed as a potential conflict of interest.
