# Supplementary material for: Predicted Bacterial Interactions Affect in Vivo Microbial Colonization Dynamics in Nematostella
Source: Front Microbiol. 2018 Apr 24;9:728. doi: 10.3389/fmicb.2018.00728 (PMC5928149; doi:10.3389/fmicb.2018.00728)
Supplement: Supplementary file 2 [file Image_1.pdf]

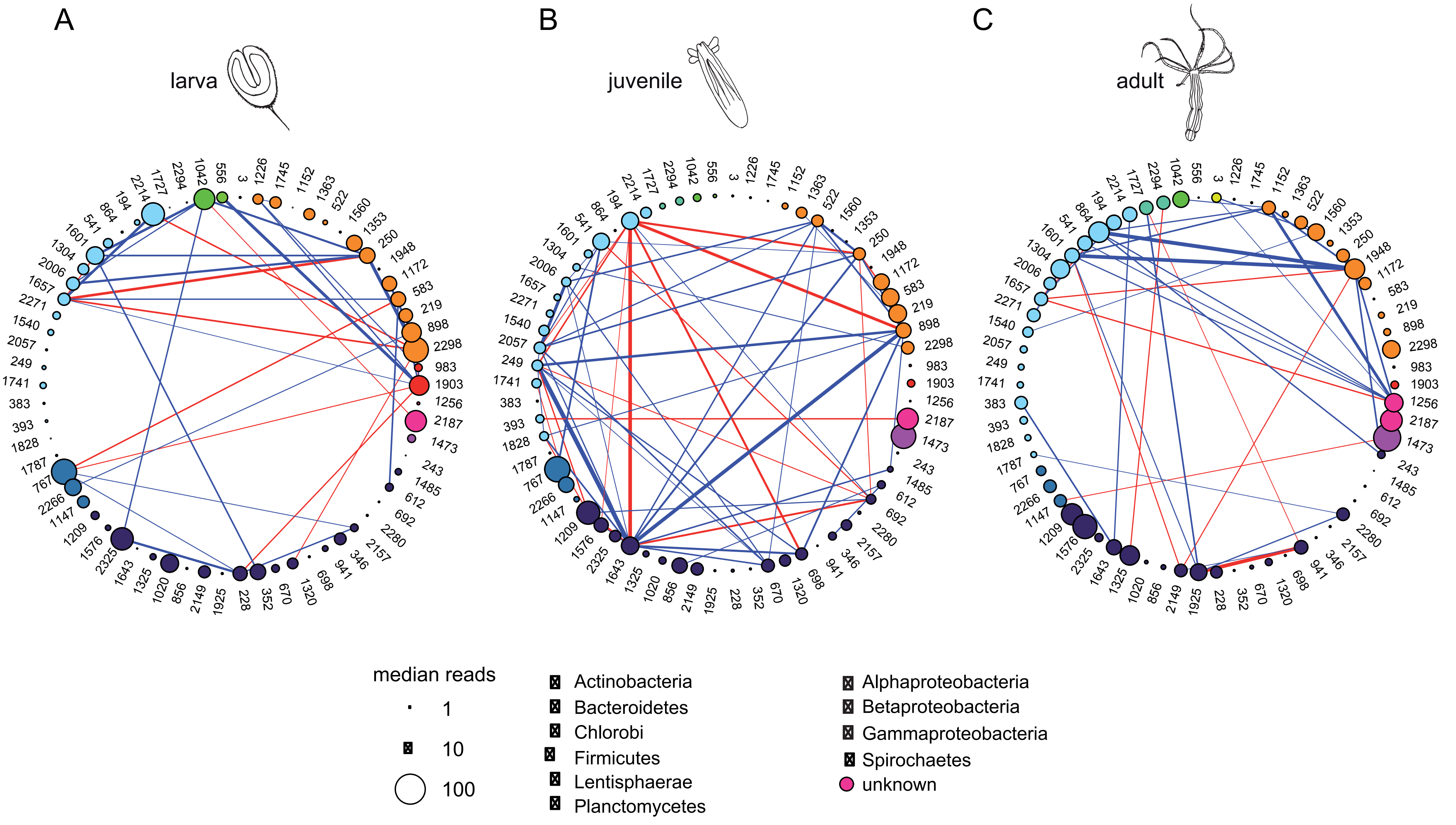

Figure S1: Microbial co-occurrence network among OTUs during the whole development of N. vectensis. Nodes (N = 66) are the OTUs involved in at least one strong interaction during the whole development or the three developmental stages; their color reflects taxonomic affiliation. The size of the nodes is proportional to the log10 of the median reads (relative abundance of the OTUs) along the whole development. OTUs are arranged by taxonomy and relative abundance (numbers refer to OTU-numbers in Mortzfeld et al., 2016). Links represent the interactions (i.e. significant co-occurrences; pseudo p-value ≤ 0.05) with absolute correlation values above 0.5. Red links are negative interactions, while blue links stand for positive interactions; the thickness of the links is proportional to the strength of the interactions.
