# Supplementary material for: Predicted Bacterial Interactions Affect in Vivo Microbial Colonization Dynamics in Nematostella
Source: Front Microbiol. 2018 Apr 24;9:728. doi: 10.3389/fmicb.2018.00728 (PMC5928149; doi:10.3389/fmicb.2018.00728)
Supplement: Supplementary file 3 [file Image_2.PDF]

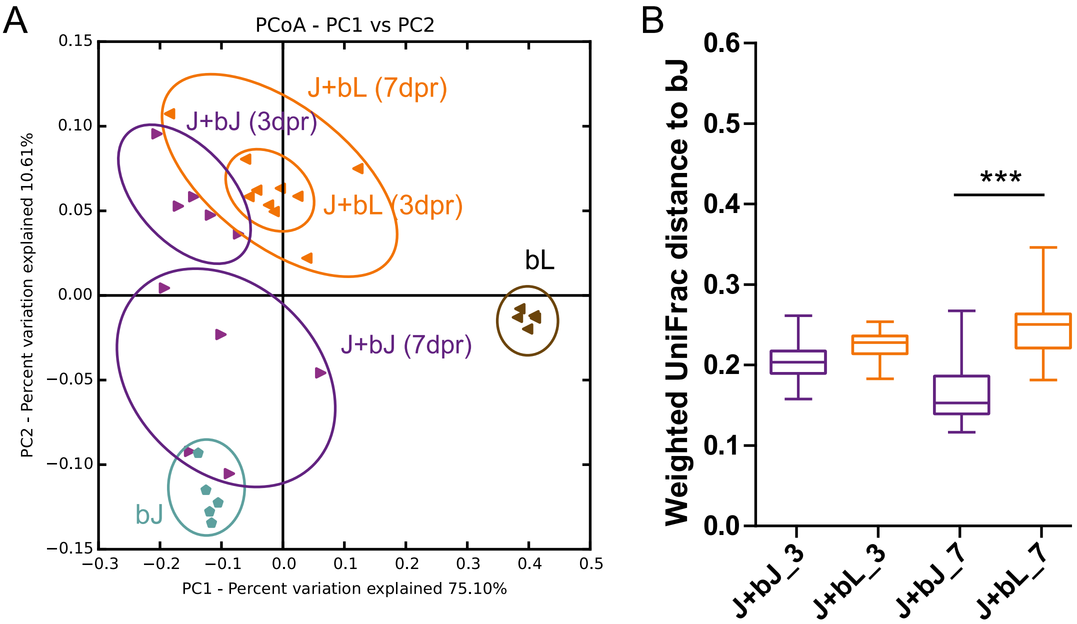

Figure S2: Juvenile polyps are recolonized differentially with larvae (bL) or juvenile bacteria (bJ). (A) Bacterial communities were clustered using PCoA of the Weighted UniFrac distance matrix. The percent variation explained by the principal coordinates is indicated at the axes. bL – source bacteria of larvae, bJ – source bacteria of juvenile polyps, J+bL – bacterial community of polyps recolonized with bL after 3dpr and 7dpr, J+bJ – bacterial community of polyps recolonized with bJ after 3dpr and 7dpr. (B) Weighted UniFrac distances to bJ after 3dpr and 7dpr. Statistical analysis was conducted using analysis of variance (ANOVA; ***$p < 0.001$, $n = 5$).
