# Supplementary material for: Predicted Bacterial Interactions Affect in Vivo Microbial Colonization Dynamics in Nematostella
Source: Front Microbiol. 2018 Apr 24;9:728. doi: 10.3389/fmicb.2018.00728 (PMC5928149; doi:10.3389/fmicb.2018.00728)
Supplement: Supplementary file 4 [file Image_3.PDF]

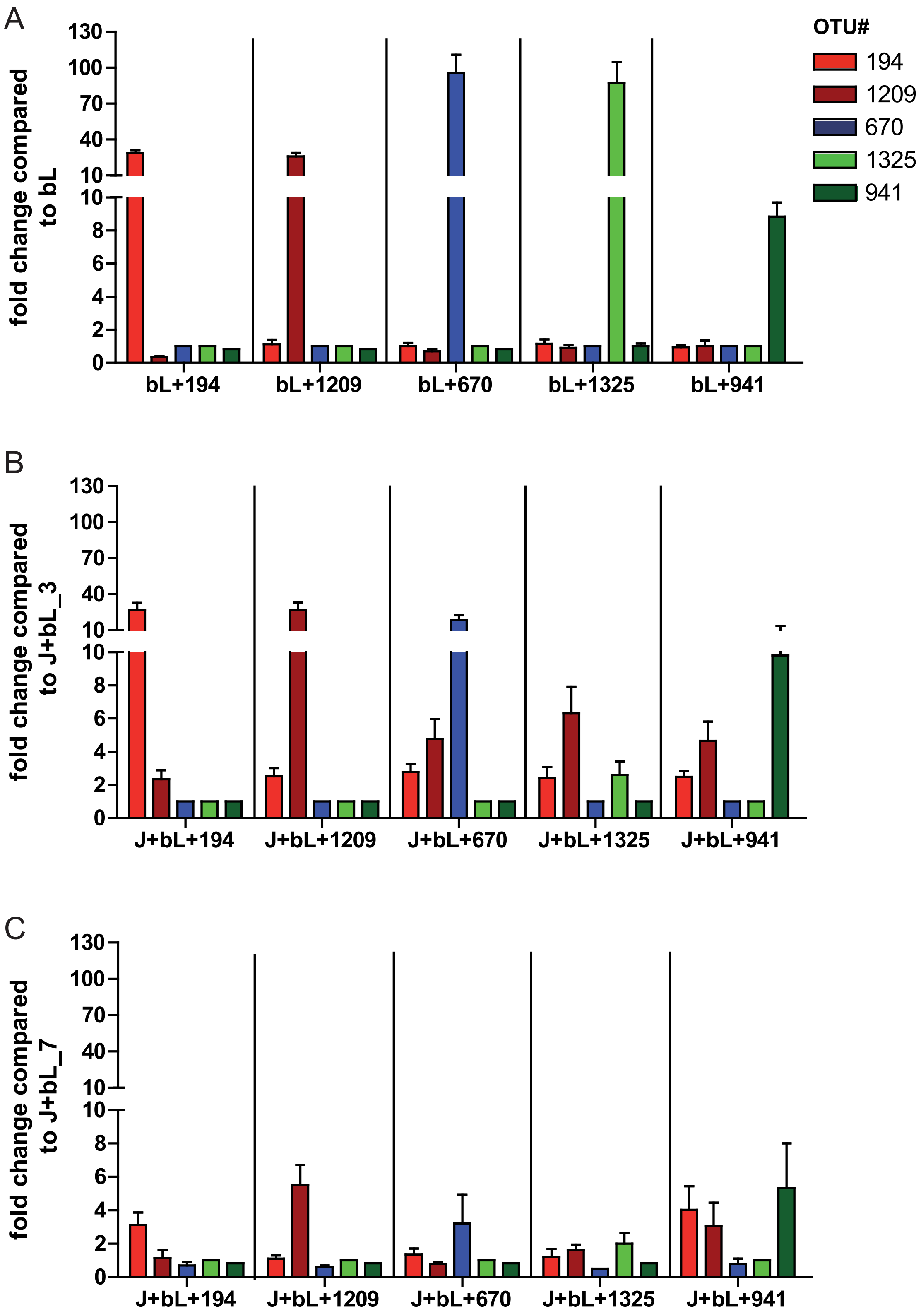

Figure S3: Overrepresentation of isolates in inocula (A), 3dpr (B) and 7dpr (C) in comparison to control colonization. Fold changes were calculated based on normalized sequencing counts of the representative OTUs (on 97% similarity).
