# Supplementary material for: Predicted Bacterial Interactions Affect in Vivo Microbial Colonization Dynamics in Nematostella
Source: Front Microbiol. 2018 Apr 24;9:728. doi: 10.3389/fmicb.2018.00728 (PMC5928149; doi:10.3389/fmicb.2018.00728)
Supplement: Supplementary file 5 [file Image_4.PDF]

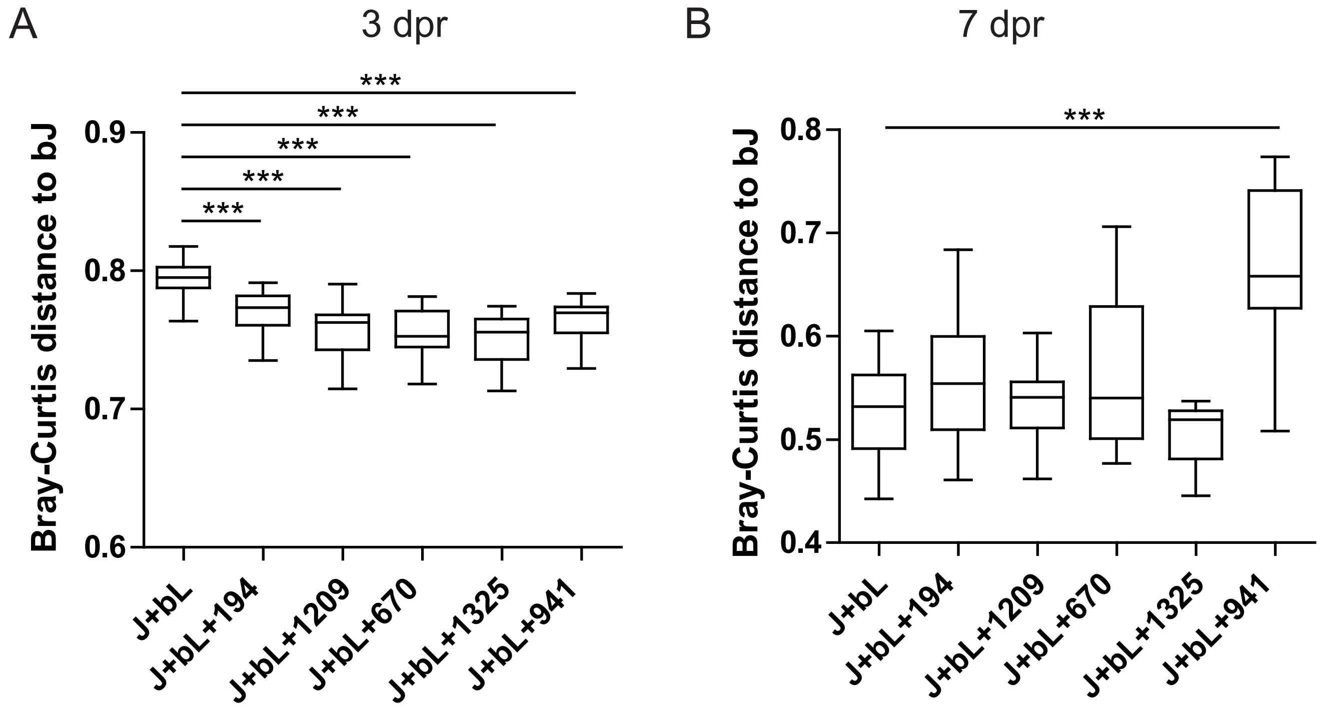

Figure S4: Bray-Curtis distances to bJ after 3dpr (A) and 7dpr (B) upon recolonization with a mixture of larval bacteria and single bacterial isolates. Statistical analysis was conducted using analysis of variance (ANOVA; ***$p < 0.001$).
